# UGT1A1^*^28 genotype and irinotecan dosage in patients with metastatic colorectal cancer: a Dutch Colorectal Cancer Group study

**DOI:** 10.1038/sj.bjc.6604461

**Published:** 2008-07-01

**Authors:** D M Kweekel, H Gelderblom, T Van der Straaten, N F Antonini, C J A Punt, H-J Guchelaar

**Affiliations:** 1 Department of Clinical Pharmacy and Toxicology, Leiden University Medical Center, Albinusdreef 2, Leiden 2333ZA, The Netherlands; 2 Department of Clinical Oncology, Leiden University Medical Center, Albinusdreef 2, Leiden 2333ZA, The Netherlands; 3 Biometrics Department, Netherlands Cancer Institute (NKI), Plesmanlaan 121, Amsterdam 1066 CX, The Netherlands; 4 Department of Oncology, Radboud University Nijmegen Medical Center, Geert Grooteplein-Zuid 10, Nijmegen 6525 GA, The Netherlands

**Keywords:** colorectal, dose, irinotecan, response, toxicity, UGT1A1^*^28

## Abstract

The aim of the study was to investigate the associations between UGT1A1^*^28 genotype and (1) response rates, (2) febrile neutropenia and (3) dose intensity in patients with metastatic colorectal cancer treated with irinotecan. UGT1A1^*^28 genotype was determined in 218 patients receiving irinotecan (either first-line therapy with capecitabine or second-line as monotherapy) for metastatic colorectal cancer. TA_7_ homozygotes receiving irinotecan combination therapy had a higher incidence of febrile neutropenia (18.2%) compared to the other genotypes (TA_6_/TA_6_ : 1.5%; TA_6_/TA_7_ : 6.5%, *P*=0.031). TA_7_ heterozygotes receiving irinotecan monotherapy also suffered more febrile neutropenia (19.4%) compared to TA_6_/TA_6_ genotype (2.2%; *P*=0.015). Response rates among genotypes were not different for both regimens: combination regimen, *P*=0.537; single-agent, *P*=0.595. TA_7_ homozygotes did not receive a lower median irinotecan dose, number of cycles (*P*-values ⩾0.25) or more frequent dose reductions compared to the other genotypes (*P*-values for trend; combination therapy: 0.62 and single-agent: 0.45). Reductions were mainly (>80%) owing to grade ⩾3 diarrhoea, not (febrile) neutropenia. TA_7_/TA_7_ patients have a higher incidence of febrile neutropenia upon irinotecan treatment, but were able to receive similar dose and number of cycles compared to other genotypes. Response rates were not significantly different.

Colorectal carcinoma is one of the most common cancers in the western world and the second largest cause of cancer-related death (Boyle and Ferlay, 2005). Approximately 50% of patients present with distant metastases either at diagnosis or during follow-up for which curative treatment is no longer possible. In the palliative setting, the median overall survival has increased from approximately 8 months without treatment to approximately 21 months using 5-fluorouracil (or its oral prodrug capecitabine), irinotecan (IRI), oxaliplatin and targeted agents (Punt, 2004). One of the most studied metabolic enzymes of the IRI pathway is uridine diphosphate glucuronosyl transferase, UGT1A1. It converts the active metabolite of IRI, SN38, to its inactive glucuronide (Iyer *et al*, 1998) and is expressed in the liver, the relative amount being dependent on the number of TA-repeats in the promoter of the gene (wild-type, TA_6_ or UGT1A1^*^28, TA_7_). The TA_7_ allele results in lower UGT1A1 expression and decreased SN-38 glucuronidation (Ando *et al*, 1998, 2002; Iyer *et al*, 1999; Paoluzzi *et al*, 2004). TA_7_/TA_7_ patients, therefore, have a higher SN38 exposure and hence an increased chance to experience toxic side effects (Iyer *et al*, 2002; Innocenti *et al*, 2004; Marcuello *et al*, 2004). One can also hypothesise that TA_6_ homozygotes may tolerate a higher IRI dosage, possibly increasing treatment benefit. Indeed, several reports show that IRI dose can be increased in a subset of patients (Merrouche *et al*, 1997; Ducreux *et al*, 1999; Ychou *et al*, 2002), and there is indirect evidence that the efficacy of IRI seems dose-dependent (Abigerges *et al*, 1995). Recently, the FDA has approved the updated Camptosar® (IRI) product labelling that recommends a reduced starting dose for TA_7_/TA_7_ patients to prevent haematological toxicity.

Our primary aim was to investigate the efficacy by genotype. Secondly, we investigated the association between UGT1A1^*^28 and febrile neutropenia as this concerns a clinically relevant complication of treatment with IRI. It leads to hospital admissions and may pose a serious medical problem, especially when occurring with diarrhoea. Thirdly, we studied the number of IRI cycles and dosage by genotype.

## Patients and methods

### Subjects

**Figure 1:**
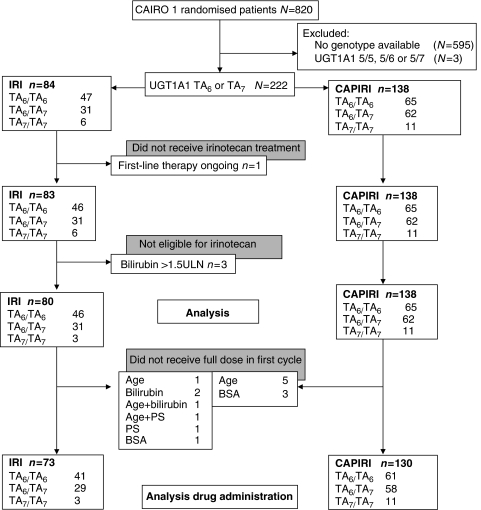
Flowchart of patients in current analysis. Abbreviations: BSA=body surface area; CAPIRI=irinotecan first-line combination therapy (250 mg m^−2^ every 3 weeks, with capecitabine); IRI=irinotecan (350 mg m^−2^ every 3 weeks) second-line single-agent therapy; PS=performance status; ULN=upper limit of normal.

Blood samples were obtained from patients enrolled in a multicentre phase III trial of the Dutch Colorectal Cancer Group (DCCG), referred to as the CAIRO study. Eligibility criteria, interim safety results (Koopman *et al*, 2006) and survival data (Koopman *et al*, 2007) have been published. Briefly, patients were allocated to regimen A or B. Regimen A consisted of first-line capecitabine (1250 mg m^−2^ day^−1^ b.i.d. on days 1–14, every 3 weeks), second-line IRI (350 mg m^−2^ day^−1^ on day 1, every 3 weeks) and third-line capecitabine plus oxaliplatin. Regimen B consisted of first-line capecitabine (1000 mg m^−2^ day^−1^ b.i.d. on days 1–14, every 3 weeks) plus IRI (250 mg ^−2^ day^−1^ on day 1, every 3 weeks: capecitabine plus IRI (CAPIRI), followed by second-line capecitabine plus oxaliplatin. Initial IRI dose of 80% in cycle 1 was recommended when: age >70 years, WHO performance status 2 and/or serum bilirubin 1.0–1.5 × upper limit of normal (ULN). If well tolerated, the dose was increased to 100% in subsequent cycles. Irinotecan dose was reduced with 25% relative to the previous cycle in case of any grade 3–4 toxicity with the exception of nausea/vomiting when adequate prophylaxis was still available. If these toxicities recurred despite dose reduction, the dose was reduced to 50% and upon next recurrence the treatment was discontinued. Diarrhoea was treated with loperamide (2 mg every 2 h, for a minimum of 12 h and a maximum treatment duration of 48 h). Prophylactic use of haematological growth factors and loperamide was not permitted. Inclusion took place from January 2003 to December 2004, and EDTA blood samples for genotyping were collected from October 2003 to March 2005 after a protocol amendment. The objective was to perform genetic association studies regarding antitumour response and toxicity. The study protocol and the amendment were approved by the local ethical committees. Patients were asked to participate in this side-study at inclusion, and written informed consent was obtained from all patients participating in the genetic association study before blood collection. DNA was obtained from 268 patients (regimen B: 141 subjects; regimen A: 127). Of the patients in regimen A, 83 continued to receive second-line therapy with IRI (Figure 1).

### Clinical evaluation

Tumour evaluation was performed by means of CT or MRI every three cycles according to RECIST (Therasse *et al*, 2000) criteria and results were blinded with respect to genotype data. Toxicity was graded according to NCI common toxicity criteria version 2.0 (Anonymous, 1998). We used febrile neutropenia and worst grade of diarrhoea experienced during treatment with IRI. Overall toxicity was defined as any grade 3 or 4 toxicity that occurred during treatment. Neutrophil counts were not routinely performed, but all patients were instructed to contact the hospital in case of fever. If so, neutrophil counts were determined. Irinotecan dose was calculated as the sum of all IRI doses (mg) and as the sum of all doses, divided by body surface area (BSA), mg m^−2^. Body surface area was calculated with the Mosteller formula, based on height and weight (at current or previous cycle). The relative dose intensity, RDI, is the dose in mg m^−2^ received divided by the protocol dose and expressed in percentage. The overall RDI is the sum of RDIs divided by the number of cycles received. A dose reduction is defined as a reduction of at least 10% compared to the previous dose. Dose reductions were analysed in patients receiving at least two doses of IRI. DNA isolation and genotyping methods are published online in the Supplementary Data file.

### Statistics

All eligible patients with UGT1A1 TA_6_/TA_6_, TA_6_/TA_7_ and TA_7_/TA_7_ genotypes who received IRI were considered for toxicity analysis. Patients were included in response analysis, if they were evaluable for response. In dosage analysis, we studied only those patients who received a full starting dose. Patients who received only one cycle were considered as having no recorded dose reduction.

Kruskal–Wallis, *χ*^2^ and (exact) Cochrane–Armitage trend tests were used to investigate the association of genotypes and patient characteristics. Logistic regression, stratified by regimen, was performed to investigate the association of UGT1A1 with the probability of febrile neutropenia. We performed bivariate analyses of UGT1A1^*^28 and a separate covariate (gender, age, site of primary tumour, prior chemotherapy, thymydilate synthase (TS) 6 bp deletion (Mandola *et al*, 2004), TS number of active repeats (Pullarkat *et al*, 2001) (as defined by Mandola (Mandola *et al*, 2003)), P-glycoproteins ABCB1 C1236T and ABCG2 C421A (Smith *et al*, 2006)) to explore a possible association of these variables with an adverse event.

All analyses were performed with SAS version 9.1.3; in all analyses, *P*-values <0.05 were considered statistically significant. We did not adjust for multiple comparisons, and therefore, this study needs to be regarded as exploratory only.

A retrospective power analysis reveals that with the given sample sizes and a genotype distribution of 45, 45 and 10% (TA_6_/TA_6_, TA_6_/TA_7_ and TA_7_/TA_7_, respectively), a Cochrane–Armitage trend test could have detected realistic differences in response rates (regimen A response rates: 6, 16 and 33% (TA_6_/TA_6_; TA_6_/TA_7_ and TA_7_/TA_7_, respectively) and regimen B: 34, 50 and 57%) with *P*=0.05 at *β*=0.8.

## Results

UGT1A1 genotype frequencies were: 50.5% (*n*=112) TA_6_ homozygotes, 41.9% (*n*=93) heterozygotes and 7.7% (*n*=17) TA_7_ homozygotes. Other genotypes found were TA_5_/TA_5_ (*n*=1), TA_5_/TA_6_ (1) and TA_5_/TA_7_ (1); these uncommon genotypes were excluded from the analysis. Patients were predominantly Caucasian. Frequencies were in Hardy–Weinberg Equilibrium and in accordance with the published results in Caucasians (Innocenti *et al*, 2004; Paoluzzi *et al*, 2004; Carlini *et al*, 2005; Toffoli *et al*, 2006). Of patients receiving first-line CAPIRI, 127 patients were evaluable for response and 138 for toxicity, and of the patients receiving second-line IRI, these were 77 and 80, respectively. Patient characteristics are shown in Table 1.

### Tumour response

Overall objective tumour response (complete (CR) and partial (PR) responses) in patients treated with CAPIRI was 47.2% (CR+PR) and 14.3% for IRI. Patients with TA_6_/TA_6_ receiving CAPIRI achieved 49.2% response, compared to 43.1% of TA_6_/TA_7_ and 62.5% of TA_7_/TA_7_ patients (*P*=0.537; Table 2). Response rates for IRI were: 15.9% TA_6_/TA_6_, and 13.3% TA_6_/TA_7_. Only stable diseases were observed in TA_7_/TA_7_ patients receiving IRI.

### Toxicity

Six of 138 patients receiving CAPIRI (5.1%) and in 7 of 80 patients receiving IRI (8.8%) experienced neutropenic fever. The incidence of febrile neutropenia in the CAPIRI regimen was 18.2% in TA_7_/TA_7_ compared to 1.5% in TA_6_/TA_6_ and 6.5% in TA_6_/TA_7_ patients (*P*=0.031, Table 2). TA_7_ homozygotes receiving CAPIRI had increased risk of 14.2 (OR 95% CI: 1.17–173) to develop neutropenic fever compared to TA_6_ homozygotes. All TA_7_/TA_7_ patients developing febrile neutropenia experienced this adverse effect in the first IRI cycle. Thirty-two of 138 patients receiving CAPIRI (23.2%) and 16 of 80 patients receiving IRI (20.0%) experienced severe diarrhoea (grade ⩾3). Of the TA_7_ homozygotes receiving CAPIRI, 36.4% experienced severe diarrhoea, as opposed to 21.5% of TA_6_ homozygotes and 22.6% of heterozygotes (*P*-value for trend: 0.43).

The incidence of febrile neutropenia was also higher in TA_7_ heterozygotes receiving IRI (*P*=0.015) relative to TA_6_/TA_6_ patients. Of the TA_6_/TA_6_ patients treated with this regimen, 2.2% experienced febrile neutropenia compared to 19.4% of TA_6_/TA_7_ patients (none of the four TA_7_/TA_7_ patients experienced this side effect). Of the TA_7_ homozygotes in this regimen, 66.7% developed grade 3 or 4 diarrhoea compared to 15.2% of TA_6_/TA_6_ and 22.6% of TA_6_/TA_7_ (*P*=0.09). The majority of severe diarrhoea episodes were not seen in cycle 1 but in subsequent cycles.

### Irinotecan dosing

As described above, we excluded from IRI dose analysis those patients who received a reduced starting dose of IRI (Figure 1). For this reason, seven patients randomised to CAPIRI and eight patients randomised to IRI were excluded. Characteristics of the patients (*n*=203) included in dosage analysis are shown in the electronic Supplementary Data file.

**Figure 2:**
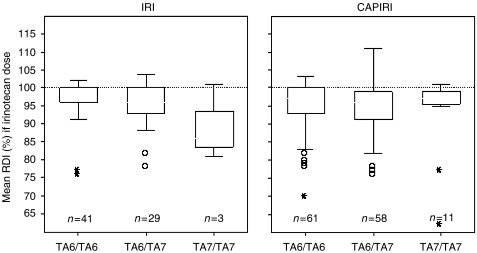
Mean RDI (%) of irinotecan dose per regimen. Boxplots of mean irinotecan dose intensity per cycle. In patients receiving either irinotecan alone or in combination with capecitabine, no statistally significant differences in relative dose intensity were observed (*P*=0.45 and *P*=0.83, respectively). Abbreviations: CAPIRI=irinotecan first-line combination therapy (250 mg m^−2^ every 3 weeks, with capecitabine); IRI=irinotecan (350 mg m^−2^ every 3 weeks) second-line single-agent therapy; 0 depicts a patient with an extreme dosage of irinotecan per cycle; ^*^depicts an outlier.

For CAPIRI, TA_7_ homozygotes did not receive a lower mean IRI dose (RDI, *P*=0.83) or median number of cycles (*P*=0.66; Table 2 and Figure 2). There was no significant difference in the total dose of IRI received over all cycles: 1.9 **g** m^−2^ (TA_7_/TA_7_) compared to 2.1 **g** m^−2^ and 2.0 **g** m^−2^ in TA_6_/TA_6_ and TA_6_/TA_7_ patients, respectively (*P*=0.51). Likewise, there were no statistically significant differences in median IRI dosage or number of cycles between UGT1A1 genotypes treated with IRI (Table 2 and Figure 2).

Individual dose adjustments in cycle 2 and subsequent cycles were made to manage serious side effects. The majority of adjustments occurred in cycles 2 and 3 for both CAPIRI and IRI. In CAPIRI, 27% of patients with the TA_7_/TA_7_ genotype received a dose reduction, compared to 18% of TA_6_/TA_6_ and 21% of TA_6_/TA_7_ (*P*-value for trend: 0.62). Reductions in cycles 2 and 3 were mainly owing to non-haematological toxicity (*n*=13; 87%), which predominantly consisted of grade ⩾3 diarrhoea. Three patients experienced febrile neutropenia or infection preceding dose reduction (one TA_6_/TA_6_, one TA_6_/TA_7_ and one TA_7_/TA_7_). Nine patients discontinued IRI before tumour evaluation at the third cycle. The discontinuation was preceded by unacceptable toxicity in six patients and included grade ⩾3 diarrhoea in all patients, but none of them experienced neutropenic fever (one TA_6_/TA_6_, four TA_6_/TA_7_ and one TA_7_/TA_7_).

In the IRI regimen, two of three TA_7_/TA_7_ (67%) patients received a dose reduction during IRI treatment, compared to 17 and 16% of TA_6_/TA_6_ and TA_6_/TA_7_, respectively. However, this difference was not statistically significant (*P*-value for trend: 0.45). Most of the dose reductions occurred in cycle 2 or 3 and 89% (*n*=8) of these were owing to gastrointestinal toxicity grades ⩾3. Two patients (22%, TA_6_/TA_6_ and TA_6_/TA_7_) experienced also febrile neutropenia or infection preceding dose reduction. Five patients (all TA_6_/TA_7_) discontinued IRI before the first scheduled tumour evaluation; of these, two patients experienced unacceptable toxicity (diarrhoea grade 3).

### Bivariate analysis of toxicity

We performed an explorative covariate analysis of patient characteristics (Table 1) and selected TS, ABCB1 and ABCG2 genotypes as they might confound associations of UGT1A1^*^28 with febrile neutropenia. We report here only those associations with *P*-values <0.05. Genotype distributions (Table 1) are in accordance with earlier publications (Cascorbi *et al*, 2001; Mandola *et al*, 2003, 2004; Lecomte *et al*, 2004; de Jong *et al*, 2004). Binary logistic regression of UGT1A1^*^28 with febrile neutropenia suggested that a performance status of 2 or a rectosigmoid tumour origin both significantly increased the risk of febrile neutropenia in the first cycle of IRI treatment (odds: 41.7, *P*=0.010 and odds: 6.5, *P*=0.030, respectively) in addition to the association of UGT1A1^*^28. A performance status of 2 increased the risk of febrile neutropenia at any time during IRI treatment 13.9-fold (*P*=0.022) and in bivariate logistic regression abolished the level of significance of the UGT1A1^*^28 association (odds for TA_7_/TA_7_: 18.5, *P*=0.128).

## Discussion

We investigated three issues concerning UGT1A1^*^28 genotype and IRI use: (1) response rates by genotype, (2) association with febrile neutropenia and (3) dosage adjustments by genotype. We found that the TA_7_ allele and the TA_7_/TA_7_ genotype are associated with an increased risk of febrile neutropenia, in patients receiving IRI and CAPIRI, respectively. Furthermore, tumour response rates were not significantly different among UGT1A1 genotypes and TA_7_/TA_7_ patients tolerated the same number of IRI cycles and dosage compared to the other genotypes.

To the best of our knowledge, this is the first study investigating the effects of UGT1A1^*^28 genotypes on IRI dose intensity using a 3-week regimen. It is also the first to study the association between UGT1A1 ^*^28 and febrile neutropenia for IRI monotherapy. In our opinion, febrile neutropenia is a more relevant clinical end point as compared to (often uncomplicated) neutropenia, as it results in hospitalisation and is potentially lethal. The current results confirm the observed trend of an increased prevalence of severe haematological toxicity among TA_7_/TA_7_ patients (Innocenti *et al*, 2004). A similar study by Toffoli *et al* (2006) shows that haematological toxicity occurring in the first cycle (but not later cycles) was related to UGT1A1^*^28 genotype. However, we found that TA_7_/TA_7_ patients receiving CAPIRI experience a higher risk of febrile neutropenia at any time during treatment as well as in the first cycle. Additionally, our results indicate that the performance status may be a strong predictor of toxicity and patients with a performance status of 2 may experience an increased risk for febrile neutropenia during IRI treatment. In bivariate analysis, performance status was associated with febrile neutropenia occurring at any cycle, whereas UGT1A1^*^28 was not. Therefore, future pharmacogenetic studies associating febrile neutropenia may consider including performance status as a covariate.

We hypothesised that TA_6_/TA_6_ patients might obtain lower response rates owing to more effective SN-38 glucuronidation. If so, response rate can possibly be improved by increasing IRI dosage in these patients. Data on UGT1A1^*^28 and response to IRI-based chemotherapy are contradicting. Carlini *et al* (2005) report a non-significant trend for an improved response rate in TA_7_ homozygotes. Ando *et al* (2000) described that TA_7_ allele carriers are at risk of developing severe toxicity by IRI and as a result, they received lower dosages. The low-dosed patients showed a non-significant trend towards better response. Higher response rates for the TA_7_/TA_7_ genotype were also found by others (Toffoli *et al*, 2006). In contrast, Marcuello *et al* (2004) reported that patients with the UGT1A1^*^28 polymorphism receiving IRI did not have a higher response rate but experienced shorter overall survival. In our study, similar to others (Liu *et al*, 2008), we found that TA_6_/TA_6_ patients had no significantly different antitumour efficacy compared to patients with other UGT1A1 genotypes.

One would expect that TA_7_/TA_7_ patients received fewer numbers of cycles and lower RDI owing to more febrile neutropenia. Indeed, a recent study with 2-week IRI (Liu *et al*, 2008) found that the need for dose reduction was associated with the TA_7_/TA_7_ genotype, but this association was not found by others (Toffoli *et al*, 2006). However, in 3-week CAPIRI, diarrhoea, and not neutropenia, may be the primary dose-limiting toxicity (Carlini *et al*, 2005; Koopman *et al*, 2006). Indeed, nearly all dose reductions in cycles 2–3 in our study were preceded only by gastrointestinal toxicity. Likewise, most patients who discontinued IRI use within the first three cycles experienced severe (grade ⩾3) diarrhoea but not neutropenic fever. However, severe diarrhoea may occur more frequently in 3-week regimens with higher IRI dosages. In these regimens, the influence of febrile neutropenia (and the UGT1A1^*^28 polymorphism) on dose intensity may be less pronounced compared to the 2-week regimen.

In conclusion, we observed that the UGT1A1^*^28 genotype is associated with an enhanced risk of febrile neutropenia but not with IRI dose reductions. However, upfront dose reduction may result in a lower incidence of febrile neutropenia in these patients.

## Figures and Tables

**Table 1:** Patient characteristics by regimen

	**IRI**					**CAPIRI**				
	**TA6**	**TA6**	**TA7**			**TA6**	**TA6**	**TA7**		
	**TA6**	**TA7**	**TA7**	**Total**		**TA6**	**TA7**	**TA7**	**Total**	
**UGT1A1**	***N*=46**	***N*=31**	***N*=3**	***N*=80**	***P*-value**	***N*=65**	***N*=62**	***N*=11**	***N*=138**	***P*-value**
*Localisation of primary tumour* (%)										
Colon	25 (54)	16 (52)	2 (67)	43 (54)	*P*=0.969^#^	33 (51)	36 (58)	7 (64)	76 (55)	*P*=0.821^#^
Rectosigmoid	5 (11)	3 (10)		8 (10)		5 (8%)	3 (5%)	1 (9)	9 (7)	
Rectal	16 (35)	12 (39)	1 (33)	29 (36)		27 (42%)	23 (37%)	3 (27)	53 (38)	
										
*Gender* (%)										
Male	25 (54)	23 (74)	3 (100)	51 (64)	*P*=0.085^#^	41 (63%)	41 (66%)	4 (36)	86 (62)	*P*=0.169^#^
Female	21 (46)	8 (26)		29 (36)		24 (37%)	21 (34%)	7 (64)	52 (38)	
										
*Age at randomisation*										
Median (range)	60.5 (46.0–78.0)	61.0 (36.0–78.0)	57.0 (48.0–75.0)	61.0 (36.0–78.0)	*P*=0.8744^$^	62.0 (44.0–81.0)	63.0 (37.0–78.0)	60.0 (46.0–74.0)	62.0 (37.0–81.0)	*P*=0.6661^$^
										
*Prior adjuvant treatment primary tumour at randomisation* (%)										
Yes	5 (11)	4 (13)	1 (33)	10 (13)	*P*=0.520^#^	7 (11)	7 (11)	3 (27)	17 (12)	*P*=0.289^#^
No	41 (89)	27 (87)	2 (67)	70 (88)						
										
*Predominant localisation of metastases at randomisation* (%)										
Liver	32 (70)	23 (74)	1 (33)	56 (70)	*P*=0.495^#^	40 (62)	48 (77)	8 (73)	96 (70)	*P*=0.147^#^
Extrahepatic	11 (24)	7 (23)	2 (67)	20 (25)		25 (38)	14 (23)	3 (27)	42 (30)	
Unknown	3 (7)	1 (3)		4 (5)						
										
*Performance status at start IRI* (%)										
0	23 (50)	18 (58)	2 (67)	43 (54)	*P*=0.857^#^	39 (60)	38 (61)	5 (45)	82 (59)	*P*=0.392^#^
1	21 (46)	12 (39)	1 (33)	34 (43)		21 (32)	22 (35)	4 (36)	47 (34)	
2	1 (2)			1 (1)		4 (6%)	2 (3)	2 (18)	8 (6)	
Missing	1 (2)	1 (3)		2 (3)		1 (2%)			1 (<1)	
										
*Bilirubin level at start IRI*										
Median (range)	10.0 (4.0–22.0)	13.0 (7.0–31.0)	27.0 (27.0–27.0)	10.5 (4.0–31.0)	*P*=0.0009^$^	8.0 (3.0–67.0)	10.0 (1.0–19.0)	13.9 (7.0–24.0)	9.0 (1.0–67.0)	*P*=0.0106^$^
										
*LDH level at start IRI*										
Median (range)	443.0 (146.0–2316.0)	436.0 (165.0–3493.0)	466.0 (310.0–604.0)	443.0 (146.0–3493.0)	*P*=0.9903^$^	410.0 (151.0–2243.0)	360.0 (119.0–3320.0)	336.5 (250.0–1213.0)	377.0 (119.0–3320.0)	*P*=0.1778^$^
										
*ABCB1* (%)										
TT	7 (15)	5 (16)	2 (67)	14 (18)	*P*=0.215^#^	7 (11)	11 (18)	3 (27)	21 (15)	*P*=0.282^#^
TC	19 (41)	14 (45)	1 (33)	34 (43)		36 (55)	34 (55)	3 (27)	73 (53)	
CC	20 (43)	12 (39)		32 (40)		22 (34)	16 (26)	5 (45)	43 (31)	
Missing							1 (2)		1 (<1)	
										
*ABCG2* (%)										
TT	36 (78)	18 (58)	3 (100)	57 (71)	*P*=0.174^#^	52 (80)	43 (69)	8 (73)	103 (75)	*P*=0.458^#^
TC	9 (20)	12 (39)		21 (26)		10 (15)	16 (26)	3 (27)	29 (21)	
CC		1 (3)		1 (1)		3 (5)	1 (2)		4 (3)	
Missing	1 (2)			1 (1)			2 (3)		2 (1)	
										
*TS number of active repeats* (%)										
1	1 (2)	2 (6)		3 (4)	*P*=0.861^#^					
2	26 (57)	19 (61)	1 (33)	46 (58)		30 (46)	33 (53)	5 (45)	68 (49)	*P*=0.306^#^
3	14 (30)	6 (19)	1 (33)	21 (26)		27 (42)	18 (29)	5 (45)	50 (36)	
4	2 (4)	2 (6)		4 (5)		3 (5)	7 (11)		10 (7)	
Missing	3 (7)	2 (6)	1 (33)	6 (8)		5 (8)	4 (6)	1 (9)	10 (7)	
										
*TS del* (%)										
del/del	5 (11)	1 (3)		6 (8)	*P*=0.488^#^	3 (5%)	10 (16)		13 (9)	*P*=0.121^#^
+6/del	12 (26)	8 (26)	2 (67)	22 (28)		28 (43)	27 (44)	4 (36)	59 (43)	
+6/+6	26 (57)	18 (58)	1 (33)	45 (56)		30 (46)	20 (32)	5 (45)	55 (40)	
Missing	3 (7)	4 (13)		7 (9)		4 (6)	5 (8)	2 (18)	11 (8)	

CAPIRI= irinotecan first-line combination therapy (250 mg m^−2^ every 3 weeks, with capecitabine); IRI: irinotecan (350 mg m^−2^ every 3 weeks) second-line single-agent therapy; LDH=lactate dehydrogenase; TS=thymydilate synthase.

^#^*χ*^2^.

^$^Kruskal–Wallis.

**Table 2:** Response, toxicity and dose during IRI treatment

	**IRI**					**CAPIRI**				
	**TA6**	**TA6**	**TA7**			**TA6**	**TA6**	**TA7**		
**UGT1A1**	**TA6**	**TA7**	**TA7**	**Total**		**TA6**	**TA7**	**TA7**	**Total**	
**Evaluable for response**	***N*=44**	***N*=30**	***N*=3**	***N*=77**	***P*-value**	***N*=61**	***N*=58**	***N*=8**	***N*=127**	***P*-value**
*Best overall response (%)*										
CR						1 (2)	3 (5)	1 (13)	5 (4)	
PR	7 (16)	4 (13)		11 (14)		29 (48)	22 (38)	4 (50)	55 (43)	
SD	27 (61)	16 (53)	3 (100)	46 (60)		27 (44)	30 (52)	2 (25)	59 (46)	
PD	10 (23)	10 (33)		20 (26)		4 (7)	3 (5)	1 (13)	8 (6)	
Response rate, %, (95% exact CI)	15.9 (6.6:30.1)	13.3 (3.8:30.7)	0.0 (0%:70.8)	14.3 (7.4:24.1)	0.595^L^	49.2 (36.1:62.3)	43.1 (30.2:56.8)	62.5 (24.5:91.5)	47.2 (38.3:56.3)	0.537^L^
Disease control, %, rate (95% exact CI)	77.3 (62.2:88.5)	66.7 (47.2:82.7)	100 (29.2:100)	74.0 (62.8:83.4)	0.240^L^	93.4 (84.1:98.2)	94.8 (85.6:98.9)	87.5 (47.3:99.7)	93.7 (88.0:97.2)	0.759^L^
										
	**TA6**	**TA6**	**TA7**			**TA6**	**TA6**	**TA7**		
**UGT1A1**	**TA6**	**TA7**	**TA7**	**Total**		**TA6**	**TA7**	**TA7**	**Total**	
**Evaluable for toxicity (grade 3–4)**	***N*=46**	***N*=31**	***N*=3**	***N*=80**	***P*-value**	***N*=65**	***N*=62**	***N*=11**	***N*=138**	***P*-value**
*Overall (%)*										
All cycles	20 (43.5)	12 (38.7)	3 (100)	35 (43.8)	Etrend 0.56	33 (50.8)	33 (53.2)	8 (72.7)	74 (53.6)	Etrend 0.35
Cycle 1	3 (6.5)	4 (12.9)	0 (0.0)	7 (8.8)	FE 0.430	3 (4.6)	6 (9.7)	1 (9.1)	10 (7.2)	Etrend 0.44
										
*Febrile neutropenia (%)*										
All cycles	1 (2.2)	6 (19.4)	0 (0.0)	7 (8.8)	FE 0.015	1 (1.5)	4 (6.5)	2 (18.2)	7 (5.1)	Etrend 0.031
Cycle 1	1 (2.2)	2 (6.5)	0 (0.0)	3 (3.8)	FE 0.561	0 (0.0)	1 (1.6)	2 (18.2)	3 (2.2)	Etrend 0.008
										
*Diarrhea*										
All cycles	7 (15.2)	7 (22.6)	2 (66.7)	16 (20.0)	Etrend 0.090	14 (21.5)	14 (22.6)	4 (36.4)	32 (23.2)	Etrend 0.43
Cycle 1	3 (6.5)	4 (12.9)	0 (0.0)	7 (8.8)	FE 0.430	3 (4.6)	6 (9.7)	1 (9.1)	10 (7.2)	Etrend 0.44
										
	**TA6**	**TA6**	**TA7**			**TA6**	**TA6**	**TA7**		
**UGT1A1**	**TA6**	**TA7**	**TA7**	**Total**		**TA6**	**TA7**	**TA7**	**Total**	
**Evaluable for dose analysis**	***N*=41**	***N*=29**	***N*=3**	***N*=73**	***P*-value**	***N*=61**	***N*=58**	***N*=11**	***N*=130**	***P*-value**
*Number of cycles*										
Median (range)	6 (3–17)	6 (1–15)	8 (4–8)	6 (1–17)	0.33^$^	9 (1–30)	9 (1–32)	9 (1–30)	9 (1–32)	0.66^$^
										
*Total dose (* *g* *)*										
Median (range)	4.4 (1.7–11.2)	3.6 (0.7–10.8)	4.0 (2.6–4.9)	4.2 (0.7–11.2)	0.39^$^	3.8 (0.4–10.7)	3.7 (0.4–14.7)	3.0 (0.5–14.7)	3.7 (0.4–14.7)	0.44^$^
										
*Total dose (* *g* * m* ^*−2*^ *)*										
Median (range)	2.1 (1.0–6.0)	2.0 (0.3–5.3)	2.3 (1.4–2.4)	2.1 (0.3–6.0)	0.25^$^	2.1 (0.3–6.2)	2.0 (0.2–8.0)	1.9 (0.2–7.6)	2.0 (0.2–8.0)	0.51^$^
										
*Dose (mg m* ^*−2*^ *) per cycle*										
Median (range)	347 (266–390)	336 (272–364)	302 (284–355)	341 (266–390)	0.45^$^	242 (84–257)	242 (190–277)	242 (156–253)	242 (84–277)	0.83^$^
Reduction of IRI after cycle 1	8	5	2	15	0.45^E^	12	13	3	28	0.62^E^
										
*Cycle of first reduction (%)*										
Cycle 2–3	4 (50)	4 (80)	1 (50)	9 (60)	0.544^L^	5 (42)	9 (69)	1 (33)	15 (54)	0.444^L^
Cycle 4–6	1 (13)	1 (20)		2 (13)	NS	4 (33)	2 (15)	1 (33)	7 (25)	NS
Cycle 7–9	1 (13)		1 (50)	2 (13)	NS	2 (17)	1 (8)	1 (33)	4 (14)	NS
Cycle ⩾10	2 (25)			2 (13)	NS	1 (8)	1 (8)		2 (7)	NS

CAPIRI=irinotecan first-line combination therapy (250 mg m^−2^ every 3 weeks, with capecitabine); CI=confidence interval; CR=complete response; E=exact; Etrend=exact-values for trend; FE=Fisher's exact; IRI=irinotecan (350 mg m^−2^ every 3 weeks) second-line single-agent therapy; L=logistic regression; NS=statistically non-significant difference; PD=progressive disease; PR=partial response; SD=stable disease.

Response is defined as CR or PR, disease control as CR, PR or SD.

*P*-values are calculated by L, Etrend, E, FE or Kruskal–Wallis.

^$^Kruskal–Wallis.
