# Supplementary material for: UGT1A1*28 genotype and irinotecan dosage in patients with metastatic colorectal cancer: a Dutch Colorectal Cancer Group study
Source: Br J Cancer. 2008 Jul 1;99(2):275–82. doi: 10.1038/sj.bjc.6604461 (PMC2480976; doi:10.1038/sj.bjc.6604461)
Supplement: Supplementary Data [file 6604461x1.doc]

**Supplementary data file**

**Genotyping**

Genomic DNA was isolated form peripheral blood cells (MagnaPure Total Nucleic Acid Isolation Kit I on MagnaPure LC (Roche Diagnostics, Mannheim, Germany)). Chromosomal DNA was quantified using Nanodrop (Isogen, Maarssen, The Netherlands) and diluted to 10 ng/μl.

UGT1A1*28 polymorphism

Primers (Table S1) and pyrosequence materials were obtained from Isogen Life Sciences (Maarssen, The Netherlands), Sepharose beads from Amersham (Uppsala, Sweden). PCR reactions were done using Hotstart PCR mastermix (Qiagen, Hilden, Germany) on MyCycle (biorad, Veenendaal, The Netherlands). Pyrosequence analysis was performed on a Pyrosequencer 96MA (Biotage, Sweden). PCR reactions were as follows: each reaction contained 10 nanograms of DNA, and 5 pmol of each PCR primer in a total of 12 microlitre. Cycle conditions were: initial denaturation for 15 minutes at 95^o^C, 35 cycles of 95^o^C -55 ^o^C -72 ^o^C each for 30 seconds, ended by 10 minutes at 72 ^o^C. The pyrosequence reactions were performed according to the manufacturer’s protocol.

TS 28bp repeat and G>C polymorphism

The number of 28 base pair repeats and the G>C polymorphism in the TS gene were determined simultaneously by means of conventional cycle sequencing (Table S1).

TS 6bp deletion, ABCB1 and ABCG2 polymorphisms

The TS 6bp deletion and single nucleotide polymorpisms (SNPs) in the ABCB1 gene at C1236T and in the ABCG2 gene at C421A were determined using TaqMan 7500 (Applied Biosystems, Nieuwerkerk aan den IJssel, The Netherlands) with a custom designed assay and according to the manufacturer’s protocol (Table S1).

Table S1. PCR and sequence primers

**Primers and probes Sequence 5'-3' Modification**

UGT1A1*28-as TGGGATCAACAGTATCTTCC 5'-Biotine

UGT1A1*28-s AAGTGAACTCCCTGCTACC

UGT1A1*28-sSeq GTATCGATTGGTTTTTGC

TS 6bp deletion-s AGCTGAGTAACACCATCGATCATG#

TS 6bp deletion-as CGTGCACGAATCCACAACAC#

TS 6bp deletion probe TGTGGTTATGAACTTTA# FAM label

TS 6bp insertion probe TGGTTATGAACTTTAAAGTTA# VIC label

TS-s 28bp repeat GTGGCTCCTGCGTTTCCCCC$

TS-as 28bp repeat TCCGAGCCGGCCACACATGGCGCGG

ABCB1 C1236T-s CACCGTCTGCCCACTCT#

ABCB1 C1236T-as GTGTCTGTGAATTGCCTTGAAGTTT#

ABCB1 C1236T CAGGTTCAGGCCCTT# FAM label

ABCB1 C1236T TTCAGGTTCAGACCCTT# VIC label

ABCG2 C421A-s CATAGTTGTTGCAAGCCGAAGAG#

ABCG2 C421A-as GGCACTCTGACGGTGAGA#

ABCG2 C421A CTGCTGAGAACTGTAAGT# FAM label

ABCG2 C421A CTGCTGAGAACTTTAAGT# VIC label

Abbreviations: # customer designed Taqman assay by Applied Biosystems; $ primer also used for sequencing; s: sense – as: antisense

| **Table S2 : Patient characteristics used for dose reduction analysis** | | | | | | | | | | | |
| --- | --- | --- | --- | --- | --- | --- | --- | --- | --- | --- | --- |
|  | | **IRI** | | | | | **CAPIRI** | | | | |
| **UGT1A1 - full startdose** | | **TA6 TA6 N= 41** | **TA6 TA7 N= 29** | **TA7 TA7 N= 3** | **Total N= 73** | **P-value** | **TA6 TA6 N= 61** | **TA6 TA7 N= 58** | **TA7 TA7 N= 11** | **Total N= 130** | **P-value** |
| Localisation primary tumor | colon | 22 (54%) | 15 (52%) | 2 (67%) | 39 (53%) | P=0.979^#^ | 31 (51%) | 34 (59%) | 7 (64%) | 72 (55%) | P=0.831^#^ |
|  | rectosigmoid | 4 (10%) | 3 (10%) |  | 7 (10%) |  | 5 (8%) | 3 (5%) | 1 (9%) | 9 (7%) |  |
|  | rectum | 15 (37%) | 11 (38%) | 1 (33%) | 27 (37%) |  | 25 (41%) | 21 (36%) | 3 (27%) | 49 (38%) |  |
| Gender | male | 20 (49%) | 21 (72%) | 3 (100%) | 44 (60%) | P=0.049^#^ | 39 (64%) | 37 (64%) | 4 (36%) | 80 (62%) | P=0.200^#^ |
|  | female | 21 (51%) | 8 (28%) |  | 29 (40%) |  | 22 (36%) | 21 (36%) | 7 (64%) | 50 (38%) |  |
| Age* | Median (range) | 60  (47-77) | 61  (36-78) | 57  (48-75) | 61  (36-78) | P=0.8597^$^ | 62  (44-81) | 63  (37-76) | 60  (46-74) | 62  (37-81) | P=0.7312^$^ |
| Prior adjuvant treatment primary tumor* | | 5 (12%) | 4 (14%) | 1 (33%) | 10 (14%) | P=0.590^#^ | 7 (11%) | 7 (12%) | 3 (27%) | 17 (13%) | P=0.343^#^ |
| Predominant localisation of metastases * | Liver | 30 (73%) | 22 (76%) | 1 (33%) | 53 (73%) | P=0.341^#^ | 39 (64%) | 45 (78%) | 8 (73%) | 92 (71%) | P=0.259^#^ |
|  | Extrahepatic | 9 (22%) | 7 (24%) | 2 (67%) | 18 (25%) |  | 22 (36%) | 13 (22%) | 3 (27%) | 38 (29%) |  |
|  | Unknown | 2 (5%) |  |  | 2(3%) |  | 37 (61%) | 37 (64%) | 5 (45%) | 79 (61%) | P=0.236^#^ |
| Performance status ** | 0 | 23 (56%) | 17 (59%) | 2 (67%) | 42 (58%) | P=0.931^#^ | 19 (31%) | 20 (34%) | 4 (36%) | 43 (33%) |  |
|  | 1 | 17 (41%) | 11 (38%) | 1 (33%) | 29 (40%) |  | 4 (7%) | 1 (2%) | 2 (18%) | 7 (5%) |  |
|  | Missing | 1 (2%) | 1 (3%) |  | 2 (3%) |  | 1 (2%) |  |  | 1 (<1%) |  |
| bilirubin level ** | Median (range) | 9.0  (4.0-22.0) | 13.0  (7.0-31.0) | 27.0  (27.0-27.0) | 10.0 (4.0-31.0) | P=0.0003^$^ | 8.0  (3.0-67.0) | 10.0  (1.0-19.0) | 13.9  (7.0-24.0) | 9.0  (1.0-67.0) | P=0.0114^$^ |
| LDH level ** | Median (range) | 432.5 (146.0-2316.0) | 415.0 (165.0-3493.0) | 466.0 (310.0-604.0) | 429.0  (146.0-3493.0) | P=0.9608^$^ | 414.5 (151.0-2243.0) | 372.5 (119.0-3320.0) | 336.5 (250.0-1213.0) | 393.5 (119.0-3320.0) | P=0.2774^$^ |

Abbreviations:

IRI2: irinotecan (350mg/m2 every 3 weeks) second line single agent therapy; CAPIRI1: irinotecan first line combination therapy (250mg/m2 every 3 weeks, with capecitabine). P-values are calculated by # chi-square; $ Kruskal-Wallis; * at randomization ** at start irinotecan.
